# “It’s My Life and It’s Now or Never”—Transplant Recipients Empowered From a Service-Dominant Logic Perspective

**DOI:** 10.3389/ti.2023.12011

**Published:** 2023-12-22

**Authors:** Wim S. Sipma, Margriet F. C. de Jong, Kees C. T. B. Ahaus

**Affiliations:** ^1^ Department of Health Services Management & Organisation, Erasmus School of Health Policy & Management, Erasmus University Rotterdam, Rotterdam, Netherlands; ^2^ Department of Nephrology, University Medical Centre Groningen, Groningen, Netherlands

**Keywords:** service-dominant logic, organ transplant, value creation, quality of life, value-based healthcare

## Abstract

Patient well-being after an organ transplant is a major outcome determinant and survival of the graft is crucial. Before surgery, patients are already informed about how they can influence their prognosis, for example by adhering to treatment advice and remaining active. Overall, effective selfmanagement of health-related issues is a major factor in successful long-term graft survival. As such, organ transplant recipients can be considered as co-producers of their own health status. However, although keeping the graft in good condition is an important factor in the patient’s well-being, it is not enough. To have a meaningful life after a solid organ transplant, patients can use their improved health status to once again enjoy time with family and friends, to travel and to return to work -in short to get back on track. Our assertion in this article is twofold. First, healthcare providers should look beyond medical support in enhancing long-term well-being. Second, organ recipients should see themselves as creators of their own well-being. To justify our argument, we use the theoretical perspective of service-dominant logic that states that patients are the true creators of real value-in-use. Or as Bon Jovi sings, “It’s my life and it’s now or never.”

## Introduction

In 2021, when the Corona virus pandemic resulted in many planned transplant operations being postponed, around 144,000 organ transplants were still performed globally. Most of these were kidney transplants (66%), followed by liver (24%), heart (6%), and lung (4%). Those 2021 data are based on the Global Observatory on Donation and Transplantation (GODT) data, produced by the WHO-ONT collaboration [1]. Organ transplants are generally the preferred treatment to improve the lives of patients suffering from organ failure [2, 3]. It is safe to say, thanks to the current high standards in organ transplant procedures, and despite the serious conditions of patients suffering from these life-threatening diseases, that, in 2021, many lives were not only saved but also improved through organ transplants. Through this, many of the organ transplant recipients and their families are now able to resume their life in a more-or-less normal way. This is an impressive worldwide achievement of all the professionals involved.

As an illustration of this, the first author (WS) of this paper is a kidney transplant recipient who has regained his well-being. He has also been a volunteer for the Dutch Kidney Patients Association for over a decade and is therefore familiar with the topic of living well after an organ transplant.

It is important that organ transplant recipients understand their personal responsibility in protecting the functioning of their new organ. In this article we distinguish two domains where patients are responsible. The first domain is “responsibility from a medical perspective,” the second is ‘about “responsibility from a personal well-being perspective.” In the first domain, healthcare professionals encourage patients to take all the necessary steps to protect the functioning of their new organ. This includes adhering to the prescribed medication, maintaining a healthy diet and having sufficient physical activity. This first domain is part of normal medical practice, also referred to as ‘the health factory’ [4], and falls within the scope of healthcare services as “diagnosing and treating illness and promoting health.” The second domain is about personal well-being, including quality of life. The sense of well-being has been associated with feelings such as experiencing positive emotions, of having self-control to a certain extent, and a sense of purpose [5]. In 2001, the World Health Organization (WHO) described well-being as a subjective state of mind that goes beyond “the mere absence of disease” and is rather “a state of complete physical, mental and social well-being” [6, 7]. Our view is that, within the personal domain, patients create their own value of living, their quality of life, and their feeling of well-being. To justify our argument, we use the theoretical framework of the service-dominant (S-D) logic. S-D logic is a holistic approach to delivering healthcare services with an active role for patients to create value. S-D logic has several similarities and differences compared to the integrated care concept and chronic care management (hereafter referred to as integrated care). In the next section we introduce S-D logic and we compare S-D logic with integrated care. Then, we discuss the relationship between S-D logic and well-being. Finally, we suggest four themes in introducing of the S-D logic in practice.

## Service-Dominant Logic and Integrated Care

**FIGURE 1:**
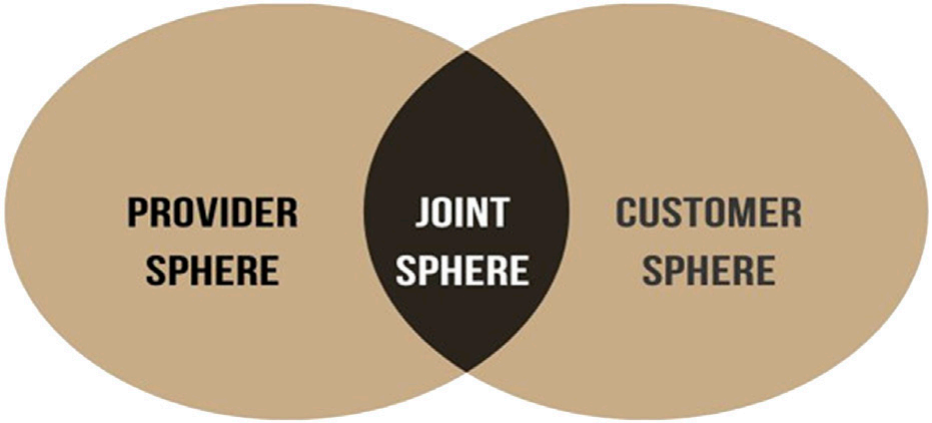
Value Creation Spheres (adapted from Grönroos and Voima [16]).

During the past decades the S-D logic framework has been developed to present a different perspective on value (co-)creation [8–10]. The traditional view in service innovation on the creation of value has been that providers deliver value to the customer, hence the service provider is the value creator [10, 11]. The S-D logic, however, distinguishes between value creation from the perspective of the provider and of the customer [10, 12–15]. According to the S-D logic, the service provider creates *potential value* in the provider sphere, whilst the provider and the customer together *co-create value* in the joint sphere. In healthcare the doctor and the patient interact in the joint sphere and co-creation is realized because doctors and patients know different things and integration of their knowledge and dialogue may lead to improved and personalized interventions [4]. Furthermore, the patient, in this case the organ recipient, is the independent creator of value-in-use (*real value)* in the customer sphere (Figure 1, adapted from Grönroos and Voima [16]). Once dismissed from the hospital after surgery the patient is on his own and, beyond self-management on health-related issues, is working hard to regain his normal life activities. This is all done in the customer sphere and highly determines the patient’s well-being.

A central theme in the S-D logic is “value-in-use” (or real value), stressing that a service in itself has no value and that value comes from its use. For transplant recipients this means that after surgery and the first recovery they resume their lives as well as possible. Patients are the creators of value and well-being in their personal lives, for instance by getting back to work. The S-D logic, with value-in-use as the core value-driver, has already been applied to healthcare [4, 17–20]. As is illustrated in the example above, S-D logic views patients as the creators of value in their private lives after having received medical care, in this case after having received a new functioning solid organ. This calls for a thorough understanding of patients’ daily environment because their home situation (customer sphere in Figure 1) is key to value creation and personal well-being. In the context of living well after an organ transplant, the S-D logic framework highlights the importance of a supportive environment for recipients since well-being is more than “just” a well-functioning new organ. A practical example in the consulting room is that, when informing patients about the possibilities of an organ transplant, the doctor mentions “you might get back to work again” (value-in-use perspective) instead of “we can transplant you with a new organ” (medical service perspective).

S-D logic can be compared with the integrated care approach. Integrated care is a well-known approach in healthcare service delivery and was developed as an answer to fragmented specialization in healthcare and especially adds value to the service of patients with chronic care needs [21–26]. Integrated care focuses on coordinated medical support to improve healthcare through the lens of patients, although it can also be considered as a multipurpose approach to develop a cost-effective, coherent care system [24, 26]. Similar to S-D logic, integrated care models are associated with interprofessional partnerships, interorganizational collaboration, patient engagement and setting patients in the heart of health service [14, 17, 27–30].

We argue that integrated care, in terms of S-D logic, is mainly focused on the joint sphere (Figure 1), the area where a variety of healthcare providers and patients interact. Where integrated care models promote a system that delivers coordinated and optimal care for and together with patients, S-D logic considers the patient as an asset, an active producer of value. We argue that this is a different way to patient involvement than described in current integrated care models. In integrated care the patient is a receiver of care whereas in the service-dominant logic approach patients are (co-)creators of value in their home environment and doctors are considered as facilitators, enabling patients to create value. We argue that this is an important and valuable addition to the role of the patient in healthcare services that aim to improve patients’ well-being. Therefore, the implementation of the S-D logic in healthcare offers a different perspective on service for patients than the paradigm that the set of medical interventions themselves deliver value, which we feel is the common premise of integrated care. A quote from an oncologist illustrates this: “Oncology practice provides treatment, but that is a fraction of the patients’ needs” [31]. To facilitate organ recipients in moving on with their lives requires supportive facilities in the patient sphere. In practice, this means that patients and care providers need to discuss what is needed for the patient to live well after an organ transplant, which specialized care within or outside the hospital can be utilized and what challenges the patient foresees. These services might go beyond the medical profession and could be offered by different professionals. To realize this, a culture of collaboration and an external orientation is needed along with patients’ awareness of their active role [10, 32]. Where patients cannot fully bear that responsibility themselves, interaction with the care provider becomes especially important. In summary, both S-D logic and integrated care promote patient centeredness. However, in our view S-D logic goes a step further by considering the patient as a resource and (co-creating) value goes beyond cooperation [33]. Value-in-use is created by the patient in the patient sphere and outside the sight of the medical profession [14, 30], which is less addressed in integrated care.

## Well-Being of Organ Transplant Recipients

If we consider the organ recipients’ well-being from the S-D logic perspective and in terms of value-in-use, we can argue that well-being is created by the organ recipients themselves after discharge from the hospital and independent of the monitoring by healthcare professionals. This creation of value by organ transplant recipients is a process that evolves out of the sight of the medical profession. During the period when patients are restoring their sense of well-being, for instance by once again socializing with their family, finding the energy to read a book, enjoying cooking, visiting cinemas and theatres, continuing their studies, reintegrating into the workplace and daring to travel again, the well-functioning of their new organ facilitates this process. In essence, this is the key message of the S-D logic: medical health services, providing diagnoses, surgery, and aftercare, should be seen as facilitators (or enablers) for patients to attain the highest possible level of well-being. The organ transplant is an indispensable starting point for patients to regain their lives, but after the operation, they have to move forward themselves. We were told of a case of a nephrologist who asked a kidney transplant patient during a regular consultation: “How are you doing?”, and the patient responded, “I think my kidney is doing well.” However, this was not what the nephrologist, who was also interested in the broader context of the patient’s well-being, meant. For the professional, the most important outcome of an organ transplant is also that organ recipients regain their lives. Although this point of view may not be groundbreaking, to serve organ recipients based on the S-D logic raises some issues. We therefore now discuss four themes related to the introduction of the S-D logic in the daily practice of organ transplant actions: the awareness that healthcare providers are facilitators, the complex process of achieving well-being, managing an S-D logic-oriented service network and rethinking value-based healthcare.

### Healthcare Providers Are Facilitators

First, transplant healthcare providers (tHCPs) should acknowledge that they are a crucial, but not the only, part of their patients’ struggles to regain their lives. While tHCPs offer potential value, this still has to be converted into value-in-use by their patients. The tHCP’s role is to facilitate patients to give meaning to their lives, and a successful complex health intervention such as an organ transplant alone is not enough. In addition to saving a life, tHCPs can have an important role in patients having a life. After providing a correct diagnosis, an organ transplant and high-quality care, the creation of real value by the organ transplant recipient continues. Here, value-in-use should be focused on well-being, which is up to the patient, possibly with support of other, possibly non-medical, facilitating health services. For instance, it is acknowledged that having a job is an important factor in a patient’s feeling of well-being [34]. Although it is certainly recognized by physicians that they can contribute to patients returning to work, it is not yet part of the collective mindset in hospitals [35]. There is a need to admit that healthcare services, even if excellent, are a part of what a patient needs: transplants are not the complete story of the patient’s journey but a necessary step that should open up a broader, more holistic, view on life after an organ transplant.

### The Complex Process of Achieving Well-Being

Second, it needs to be recognized that creating well-being is a process that involves various actors surrounding the sphere of the patient, and that achieving patients’ psychological ownership of their well-being is complex [36]. Further, the development of services to support the creation of well-being affects the entire healthcare service system. Well-being is multidimensional and is influenced by many aspects such as health, employment, income, and relationships [37] and, given that these influences may change over time, it is not an easy task for tHCPs to identify their role in this complexity. For instance, it is suggested that recovering and regaining quality of life after a liver transplant is influenced by the occurrence of depression before a transplant [38], illustrating the complexity of achieving well-being. We can picture two roles for tHCPs beyond their core medical task: a) to motivate the organ transplant recipient to take personal responsibility for the creation of well-being; and b) to have some knowledge on related services that might help patients who are confronted with issues such as loneliness or loss of income or job.

### Managing an S-D Logic-Oriented Service Network Partnership

Third, management has the responsibility to make decisions on the scope of services to be offered by the organization, either at the unit (department) or at the organization (hospital) level. The scope of services that are offered beyond medical care should be discussed. These extended services should aim to support organ recipients in creating well-being in their daily lives. For instance, since employment is considered an important influence on well-being [39, 40], a possible service would be to support work retention. Similarly, budget coaching and relationship coaching are possible additional services because coping with chronic illness may affect income and relationships [41, 42]. There is no need for hospitals to offer these extended health services themselves, there may be other more suitable providers to turn to for support. Here, the role of the hospital would be to connect with external providers and align the provided service levels. The S-D logic refers to these extended health services, offering collaborative care to realize a holistic service approach, as the service ecosystem [18, 43]. This ecosystem is characterized by multiple actors, most likely from different organizations, that together create a context to enable value creation by the organ recipient. Although moving a hospital to an S-D logic-oriented service network partnership is a managerial challenge [32], we believe that transplant recipients may benefit from this transition.

### Rethinking Value-Based Healthcare

Fourth, when adopting the value-in-use paradigm, there is a need to rethink the concept of value-based healthcare (VBHC). Value-based healthcare focuses on ‘what matters most to patients’ and relates these outcomes to costs [44], although what this means in practice is somewhat unclear [45]. In practice, the concept of VBHC focuses mainly on the direct healthcare context and less on the broader context of well-being as described in this paper. We notice that the majority of quality metrics in solid organ transplantation focuses on safety and effectiveness although a plea is made for more patient involvement and a focus on what really matters to patients in an broader healthcare context [46]. Patient reported outcome measures (PROMs) are considered to represent the patient’s perspective but are hardly used in the clinical practice of kidney transplants [47]. However, the benefits of PROMs are mainly described in terms of better doctor-patient communication and improved healthcare self-management of patients [48] thus leaving out the possibilities of value creation in the patient’s sphere. We can imagine that in the future PROMs, being the backbone of value-based healthcare (VBHC), evolve and take the daily life of transplant recipients into consideration. In our view, accepting the paradigm that healthcare organizations are the enablers of value creation, and that organ transplant recipients are the creators of value-in-use, would lead to a more prominent role for patients’ self-determination [49]. Whereas VBHC is aiming to create value *for* the patient, we argue that value is created *with and by* the patient. On this basis, we would urge the intensification of patient involvement in designing healthcare services on the grounds that patients are the co-creators of value in healthcare and well-being [50–54].

## Conclusion

The well-being of organ transplant recipients is not only realized through good medical practice. Keeping the graft in good condition and sustaining long-term graft survival are important facilitators for organ recipients to regain their lives. Embracing the paradigm of S-D logic by the professional transplant community may lead to a supportive healthcare service system that in addition to high medical quality transplants, also takes into consideration the capabilities of transplant recipients to regain their daily life, in all its aspects. After all, transplant recipients could sing along with Bon Jovi “It’s my life and it’s now or never.”

## Data Availability

The original contributions presented in the study are included in the article/supplementary material, further inquiries can be directed to the corresponding author.
